# Platoon dispersion model in the mixed traffic environment

**DOI:** 10.1038/s41598-023-45539-9

**Published:** 2023-11-03

**Authors:** Wei Cheng, Ye-xiao Chen

**Affiliations:** Faculty of Transportation Engineering, School of Traffic Engineering, Kunming University of Science and Technology, Kunming, 650000 China

**Keywords:** Environmental social sciences, Engineering

## Abstract

To describe the phenomenon of platoon dispersion in the mixed traffic environment and provide a theoretical basis for signal timing optimization in the mixed traffic environment. Based on Robertson’s platoon dispersion model, this paper fully considers the characteristics of mixed traffic conditions, and models the platoon dispersion in the mixed traffic environment. Firstly, considering the influence of the percentage of non-motor vehicles on the platoon dispersion, the functional relationship between the percentage of non-motor vehicles and the parameters of the platoon dispersion model is derived, and the parameters of the Robertson platoon dispersion model are calibrated. Secondly, through the data collected from Zitai Road in the city of Kunming, the required model parameters are calculated, and the arrival flow rate distribution downstream of the intersection is obtained. Finally, according to the measured data, the proposed model is compared with the classical Robertson model. The results show that compared with the Robertson model, the average prediction mean square error of the proposed model is reduced by 5.89%, which can better describe the dispersion law of the mixed traffic under the influence of different percentage of non-motorized vehicles.

## Introduction

On urban roads, due to the existence of signal control, the traffic flow will be divided into a group of convoys from the upstream intersection. At the same time, in the process of driving, due to the differences of driving behaviors and operating conditions in the fleet, the fleet will be continuously elongated in the process of driving downstream, which is called ‘discrete phenomenon’. Through the study of the fleet discrete model, it will help to optimize the coordinated control of urban road traffic signal. At present, many scholars have carried out in-depth research on this.

In 1956, PACEY^1^ first proposed a discrete model of traffic density, which assumes that the vehicle travel speed obeys the normal distribution. Vincent et al.^2^ believed that vehicles's travel time between the two sections of the road will be different, and the travel time is distributed according to the Geometric distribution. Based on Pacey's normal distribution model, Grace^3^ proposed a discrete model to describe the change of traffic density. Based on the traffic flow data collected by Hillier et al.^5^, Robertson^4^ proposed a discrete model of traffic flow based on the shift geometry distribution of vehicle travel time. This model uses fast iterative calculation, so it is widely used in TRANSYT-7F^4^, SCOOT^6^, SATURN^7^ and TRAFLO^8^ traffic simulation and control systems. When applying the Robertson model, Yu et al.^9^ proposed an alternative method to calibrate these parameters in the discrete distribution model. They also established three equations to calibrate, and smooth factors based on the average link travel time and its standard deviation. Yu also found that even on the same street, the standard deviation of the fleet dispersion parameters varies with the travel time of the road section, so the fleet dispersion parameters must be calibrated according to the specific location.

Many Chinese scholars have also studied the discrete model of the fleet. Based on the actual survey data of Changchun City, Wang et al.^10^ established a fleet discrete model based on non-transform normal distribution, and proposed a traffic feedback mechanism prediction method, which has higher prediction accuracy. Bie et al.^11^ also discussed the influence of the number of lanes on the platoon dispersion of traffic flow under low friction conditions, and recalibrated the platoon dispersion coefficient with the data of different lane number sections. Considering the boundedness of vehicle speed, Wu et al.^12^ proposed to use truncated normal distribution and truncated logarithmic normal distribution to fit the distribution of vehicle speed and travel time, and established a fleet discrete model from the perspective of fleet flow and fleet density. Based on the field data collection of traffic flow sections in Beijing, Yu^13^ studied the fleet discrete model proposed by Robertson, and obtained the best statistical time of the survey data in the model. Based on the Robertson model, Yao et al.^14,15^ constructed a heterogeneous traffic platoon discrete model based on the characteristics of traffic flow under heterogeneous traffic flow conditions to achieve real-time traffic signal control optimization. At the same time, in order to solve the problem that the parameters of the traditional platoon discrete model cannot reflect the real-time variation characteristics of traffic flow, Yao et al.^16^ used the real-time travel time data in the Internet of vehicles to dynamically estimate the model parameters, and on this basis, constructed a dynamic platoon flow discrete model with travel time subject to truncated normal distribution. Shen et al.^17^ established a dynamic platoon discrete model, which can detect the change of traffic flow in the cross section of the road, predict the evolution of traffic flow, and further generate a signal timing plan. In order to reduce the delay at signalized intersections, Liu et al.^18^ derived a new delay model based on the degree of freedom and randomness of speed changes and considering the delay caused by the discrete behavior of the platoon.

**Figure 1:**
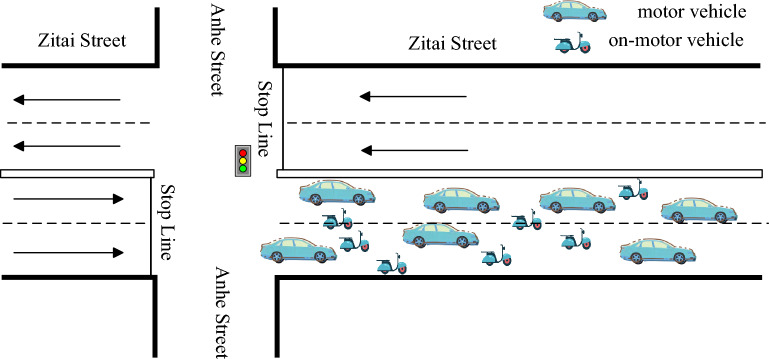
Mixed traffic.

With the development of the current society, the number of motor vehicles is rising, but non-motor vehicles as a traditional means of transport, the user group is still very large. Therefore, on some urban roads, the mixed driving of motor vehicles and non-motor vehicles has become a typical traffic phenomenon, as shown in Fig. 1. After on-the-spot investigation, it is found that the urban roads in many parts of the country are only motor vehicles, but there is no lane planning for non-motor vehicles. For example, 55% of the roads in Qilin District, Qujing City, Yunnan Province are unable to plan special non-motor vehicles due to limited space. In the process of vehicle driving, non-motor vehicles will be mixed with motor vehicles, which not only causes great hidden dangers to road traffic safety, but also greatly reduces the road capacity. Therefore, based on the traditional Robertson model, this paper considers the influence of the proportion of non-motor vehicles on the model parameters, and uses the actual survey data to estimate the model parameters. The calculation results are compared with the actual data, which proves that the model has high accuracy.

## Robertson platoon dispersion model

Robertson model is the most widely used discrete distribution model, which is one of the important components of the adaptive signal control system TRANSYT^4^. The basic assumption of the model is that the vehicle travel time obeys the shift geometric distribution, and the downstream arrival flow distribution is predicted by the upstream departure flow distribution. In the relevant literature^4^, the model expression given is as follows:

1 $$ q{\prime} \left( t \right) = F \cdot q\left( {t - T} \right) + \left( {1 - F} \right) \cdot q{\prime} \left( {t - 1} \right) $$

2 $$ F = \frac{1}{{1 + \alpha \cdot \beta \cdot T_{a} }} $$

In the formula :$q{\prime} (t)$ is the flow to the downstream intersection within the time interval $t$(veh/h); $q(t)$ is the number of vehicles leaving the upstream intersection within the time interval $t$;$T$ is lag time, take $\beta \cdot T_{a}$; $\alpha$ is the dispersion coefficient of the fleet; $\beta$ is the travel time coefficient; $F$ is a smoothing factor, which represents the coefficient of the dispersion degree of the fleet; $T_{a}$ is the average travel time between upstream and downstream intersections.

In the application of the Robertson model, most studies focus on the calibration of parameters $\alpha$ and $\beta$. The different values of $\alpha$ and $\beta$ determine the arrival mode of vehicles approaching the intersection, which significantly affects the optimal timing of the signal. Therefore, proper calibration of $\alpha$ and $\beta$ values is conducive to optimizing traffic signal control and intersection management. Research has shown that the TRANSYT-7F^4^ user guide provides $\alpha$ and $\beta$ values, where $\beta$ is a fixed value of 0.8 and $\alpha$ is one of three values of 0.25, 0.35 or 0.5, but it is not applicable to many road scenarios, especially in road environments where the proportion of non-motor vehicles is too high.

## Model parameters calibration

### Data collection

**Figure 2:**
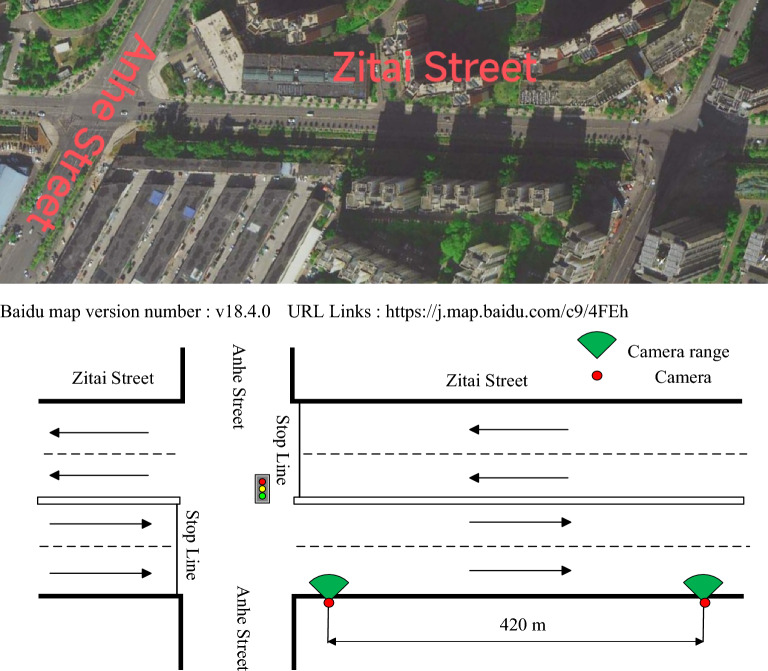
Diagram of survey road segment.

In order to calibrate the Robertson discrete distribution model, this paper selects Zitai Road in Kunming for data acquisition, studies the discrete situation on the road section, as shown in Fig. 2, briefly introduces the basic situation of the road section. The survey area is composed of an intersection and a section. There is no bus stop between the sections, and the section is bidirectional 4 lanes. The reason for choosing this section is that there are neighborhoods and schools near this section, so there are more non-motor vehicles driving on the section, and the proportion of non-motor vehicles is high. The whole section presents the characteristics of mixed traffic flow, which is very suitable for studying the discrete model of the fleet under different proportions of non-motor vehicles.

Data collection uses license plate method. When the discrete process of the vehicle fleet on the research site is recorded, all cameras are set to the same starting time. By shooting the upstream intersection exit location and downstream section of the vehicle through the video, and then data processing. The time of each vehicle passing through each section is obtained, and then the time difference of each vehicle passing through the section is obtained. The travel time of each vehicle can be obtained, and the standard deviation of the corresponding road travel time can also be calculated.

### Model parametric analysis

Yu et al.^9^ proposed a method to calibrate the discrete parameters of the fleet based on the link travel time. Based on the average road travel time and its standard deviation, three equations are established to calibrate $\alpha$, $\beta$ and $F$ smoothing coefficients. This method can take into account the impact of various traffic conditions, such as the impact of the proportion of non-motorized vehicles at signalized intersections on the fleet dispersion. The formulas used to calibrate $\alpha$, $\beta$ and $F$ smoothing coefficients are as follows:

3 $$ \alpha = \frac{{ - 1 + \sqrt {1 + 4\sigma^{2} } }}{{2T + 1 - \sqrt {1 + 4\sigma^{2} } }} $$

4 $$ \beta = \frac{1}{1 + \alpha } $$

5 $$ F = \frac{{ - 1 + \sqrt {1 + 4\sigma^{2} } }}{{2\sigma^{2} }} $$

In the formula, $\sigma$ is the standard deviation of the travel time of the vehicle section in each fleet.

**Figure 3:**
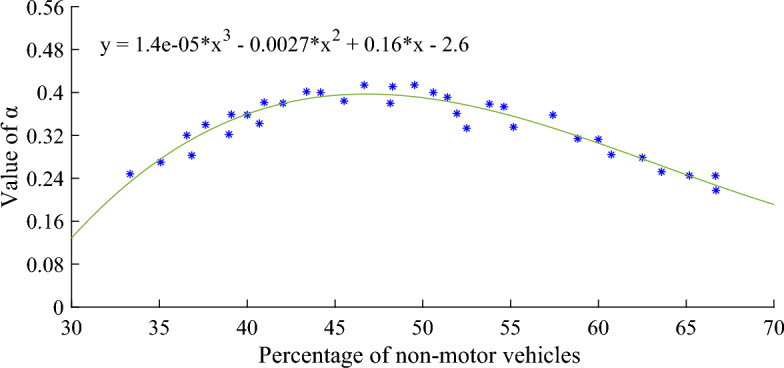
Relationship between percentage of non-motor vehicles and platoon dispersion factor $\alpha$.

The programs written in MATLAB calculate $\alpha$, $\beta$ and $F$ according to Eqs. (3), (4) and (5). The proportion of non-motor vehicles in each platoon was extracted from the field data, ranging from 33.33 to 66.70%. In order to analyze the influence of the proportion of non-motor vehicles on the discrete parameters of the fleet, regression analysis was carried out. Figure 3 shows the relationship between the proportion of non-motor vehicles and the fleet dispersion coefficient $\alpha$.

The regression relationship between the proportion of non-motor vehicles and the discrete coefficient $\alpha$ of the fleet is shown in Eq. (6):

6 $$ \alpha { = 1}{\text{.4}} \cdot {10}^{{{ - }5}} x^{3} - 0.0027x^{2} { + }0.16x - 2.6 $$

where $x$ is the proportion of non-motor vehicles. It can be seen from Fig. 3 that with the increasing proportion of non-motorized vehicles, the discrete coefficient $\alpha$ of the fleet increases rapidly. When the proportion of non-motor vehicles increased to 46.31%, the dispersion coefficient $\alpha$ reached the maximum 0.4096. However, with the increase of the proportion of non-motor vehicles, the dispersion coefficient $\alpha$ began to decrease slowly. Therefore, it can be concluded that it is necessary to continuously adjust the discrete coefficient $\alpha$ of the fleet in order to optimize the signal control of urban intersections under different road traffic conditions.

**Figure 4:**
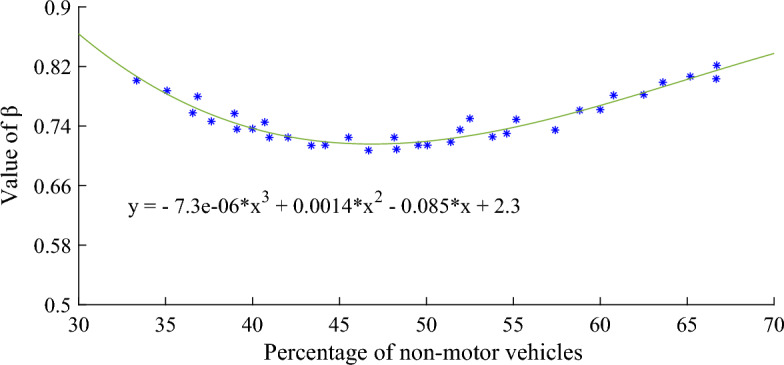
Relationship between percentage of non-motor vehicles and travel time factor $\beta$.

The relationship between the proportion of non-motor vehicles and the travel time coefficient $\beta$ is shown in Fig. 4, and the corresponding mathematical relationship is shown in Eq. (7):

7 $$ \beta { = } - {7}{\text{.3}} \cdot {10}^{{{ - }6}} x^{3} + 0.0014x^{2} - 0.085x + 2.3 $$

In the TRANSYT-7F User Guide, it is recommended that travel time factor $\beta$ take a fixed value of 0.8, but as shown in Fig. 4, travel time factor $\beta$ decreases as the proportion of non-motor vehicles increases. The reason is that the increase of non-motorized vehicles has caused great interference to the driving of motor vehicles, and the driving speed of vehicles will slow down, which requires more time to reach the downstream intersection. When the proportion of non-motor vehicles increases to 47.26%, the travel time coefficient $\beta$ reaches the minimum 0.7158. But as the proportion of non-motor vehicles continues to increase, travel time coefficient $\beta$ began to rise slowly. According to the observation of road traffic flow, when the proportion of non-motor vehicles increases to about 60%, non-motor vehicles become the main body of road traffic operation. Non-motor vehicle owners are no longer content to rely solely on the right side of the road, but arbitrarily interspersed across the lane separation line, squeezing the vehicle' s driving space. Motor vehicle owners for driving safety considerations, will also intentionally avoid non-motor vehicles.

The smoothing coefficient F will also be affected by the proportion of non-motor vehicles. The mathematical relationship between the two is shown in Eq. (8):

8 $$ F{ = } - {3}{\text{.7}} \cdot {10}^{ - 8} x^{3} + 0.00015x^{2} - 0.015x + 0.43 $$

**Figure 5:**
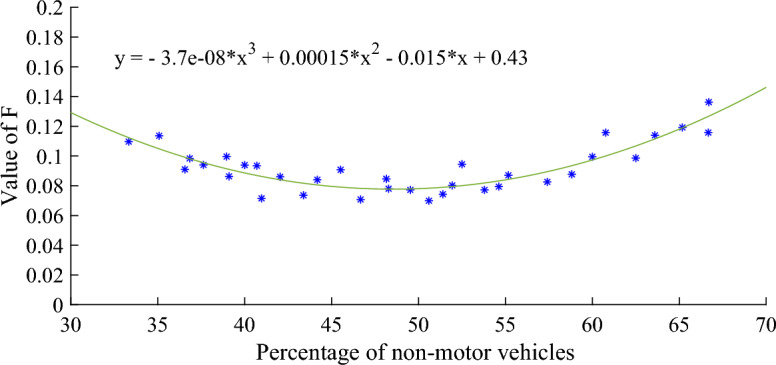
Relationship between percentage of non-motor vehicles and smoothing factor $F$.

As shown in Fig. 5, when the proportion of non-motorized vehicles increases, the smoothing coefficient $F$ decreases and the discrete speed of the fleet decreases. When the proportion of non-motor vehicles increases to 50.96%, the smoothing coefficient $F$ reaches the minimum 0.0781. However, when the proportion of non-motor vehicles continues to increase, the smoothing coefficient F began to increase. Therefore, it can be seen that the non-motorized vehicles in the fleet will affect the dispersion of the fleet in the mixed traffic environment.

The relationship between $\alpha$, $\beta$ and $F$ shown in Figs. 3, 4 and 5 can be explained as follows. According to Robertson model and related formulas, the discrete coefficient $\alpha$ is inversely proportional to the travel time coefficient $\beta$ and the smoothing coefficient $F$, and $F$ is proportional to the discrete degree of the fleet. Therefore, $\alpha$ is inversely proportional to the dispersion of the fleet. In Fig. 3, the relationship between the proportion of non-motor vehicles and the fleet dispersion coefficient $\alpha$ is taken as an example. When the proportion of non-motor vehicles is low, the interference of non-motor vehicles on the road is small, the driver can drive at the desired speed, and the speed difference between vehicles is large, resulting in high dispersion between vehicles, so the value of $\alpha$ is small. As the proportion of non-motor vehicles continues to increase, the interference between non-motor vehicles and motor vehicles begins to intensify, and the driving between them will be restricted by each other. Under such traffic conditions, the movement of traffic flow tends to be stable, the dispersion of the fleet decreases, and the value of $\alpha$ begins to increase. When the proportion of non-motorized vehicles further increases, the interference of non-motorized vehicles on motor vehicles intensifies. In order to achieve the desired speed, some non-motorized vehicle owners frequently accelerate or decelerate, arbitrarily interspersing between motor vehicles, squeezing the driving space of motor vehicles. And motor vehicle drivers, for traffic safety considerations, will consciously reasonable avoidance of non-motor vehicles. The traffic state of the whole section becomes more unstable, so the dispersion of the fleet increases again and the value of $\alpha$ begins to decrease.

**Figure 6:**
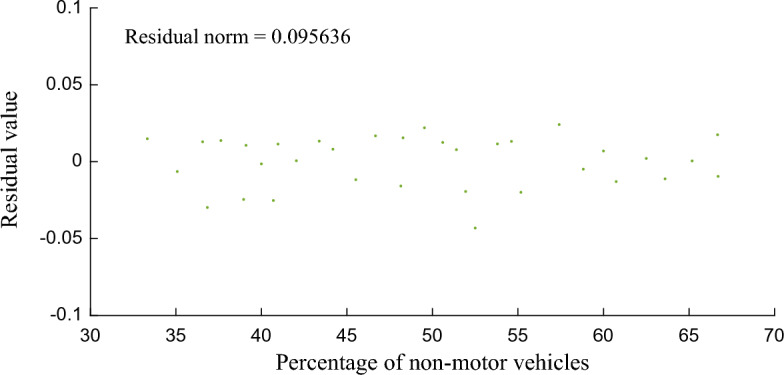
Residual value between percentage of non-motor vehicles and platoon dispersion factor $\alpha$.

**Figure 7:**
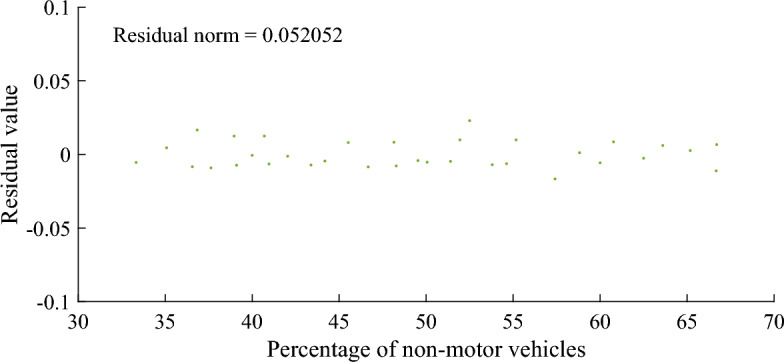
Residual value between percentage of non-motor vehicles and travel time factor $\beta$.

**Figure 8:**
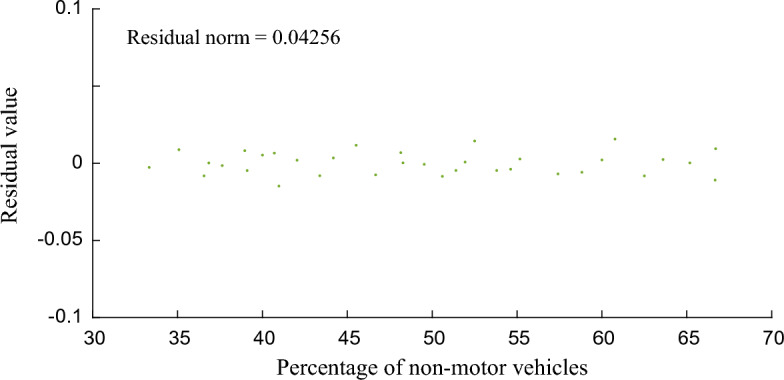
Residual value between percentage of non-motor vehicles and smoothing factor $F$.

The smaller the residual norm, the better the fitting effect and the higher the accuracy. Figures 6, 7 and 8 show that the residual norm of the proportion of non-motor vehicles and the three parameters of the fleet discrete model are 0.0956, 0.0521 and 0.0426, respectively, indicating a good correlation between the two. Therefore, the mathematical relationship established by Eqs. (6), (7) and (8) can well determine the impact of the proportion of non-motor vehicles on the fleet dispersion. At the same time, it can be found that when the proportion of non-motor vehicles is close to 50%, the impact on the whole fleet dispersion is the largest. Therefore, in the optimization design of signal timing in mixed traffic environment, it is necessary to focus on the discrete arrival effect of different proportions of non-motorized vehicles.

## Model validation

### Parameter estimation

In order to verify the effectiveness of the model, this paper selects multiple sets of data from the actual scene, and compares the discrete parameters of the fleet obtained by the model with the actual observations. Specific model parameter values can be estimated according to the formula (5), (6), (7) and (8), where $F_{n}$ is the smoothing coefficient obtained by this model,$F_{m}$ is the smoothing coefficient obtained by Robertson model, the results are shown in Table 1.

**Table 1:** The parameters of model.

Scenario	Sample size	Non-motor vehicles (%)	$\alpha$	$\beta$	*T*_*a*_	*T*	$F_{n}$	$F_{m}$
1	420	44.72	0.3940	0.7173	39.42	28.28	0.0818	0.0689
2	420	56.35	0.3435	0.7447	40.51	30.17	0.0852	0.0766
3	420	65.00	0.2474	0.8014	41.39	33.17	0.1165	0.0901

### The arrival flow rate distribution at downstream intersection

**Figure 9:**
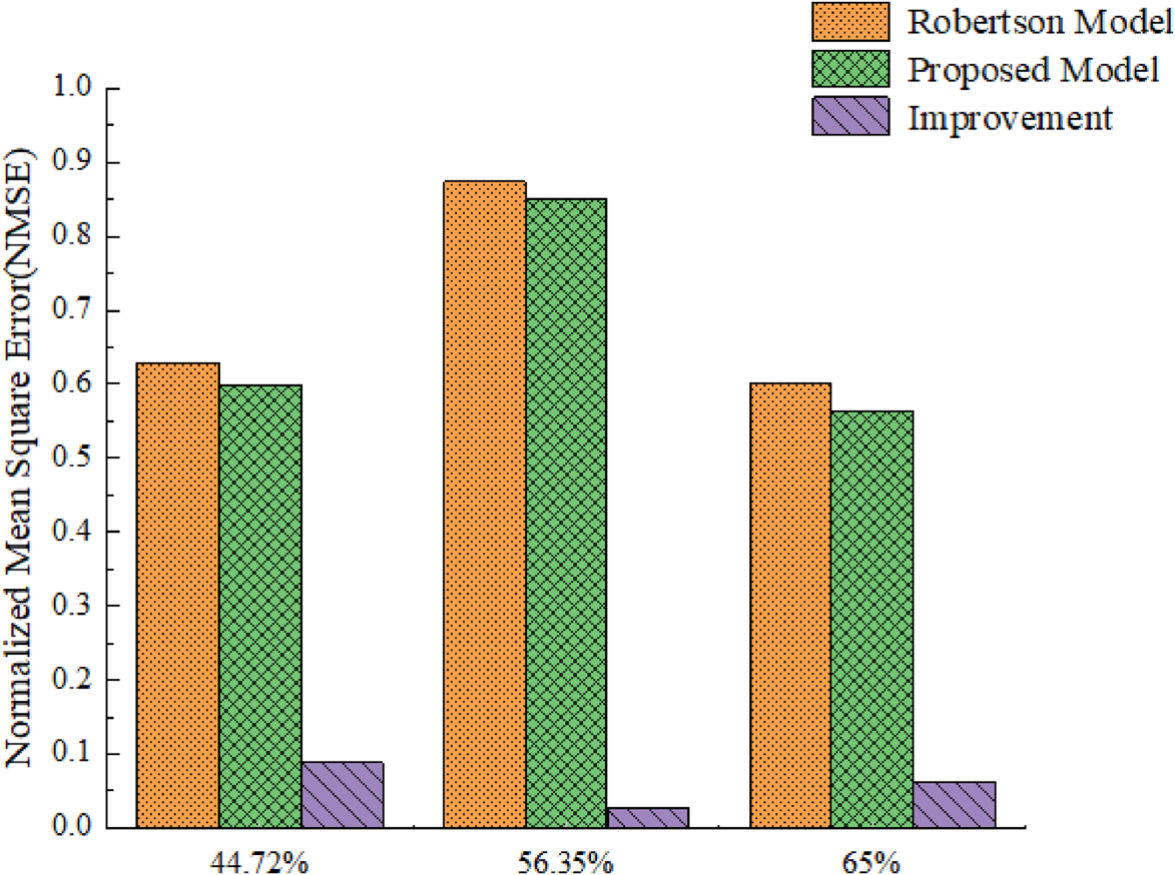
The normalized mean square error of the two models.

The model parameters and survey data obtained in Table 1 are introduced into this model and Robertson model to obtain the flow distribution of the vehicle fleet reaching the downstream intersection. The actual downstream arrival flow distribution can be obtained by observation. At the same time, the accuracy and effectiveness of this model can be verified by comparing the calculation results of this model and Robertson model with the actual data of the survey. As shown in Fig. 9, the abscissa refers to the travel time of each vehicle in the convoy to the downstream observation section, and the ordinate refers to the flow rate of the convoy to the downstream observation section.

### Comparison and analysis of these models

In the process of model validation, the prediction results can be compared with the actual vehicle arrival results to evaluate the accuracy of the model. In this paper, the normalized mean square error (NMSE) is used for quantitative measurement, and this method is used to evaluate the accuracy of the model. The formula (9) is as follows.

9 $$ NMSE = \frac{1}{n}\sum\limits_{i = 1}^{n} {\frac{{\left( {q_{{_{oi} }} - q_{ei} } \right)^{2} }}{{\overline{q}_{o} \cdot \overline{q}_{e} }}} $$

where $n$ is the predicted number of cycles; $q_{oi}$ is the actual sample arrival flow value observed at time $i$; $q_{ei}$ is the predicted sample arrival traffic value at $i$ time; $\overline{q}_{o}$ is the average of observed flow; $\overline{q}_{e}$ is the average of the estimated flows.

In the three groups of comparative cases, the values of NMSE are shown in Table 2.

**Table 2:** The normalized mean square error.

Scenario	Non-motor vehicles (%)	*NMSE *(mixed)	*NMS E*(Robertson)	Improvement (%)
1	44.72	0.5973	0.6289	8.83
2	56.35	0.8511	0.8734	2.55
3	65.00	0.5630	0.6008	6.29

**Figure 10:**
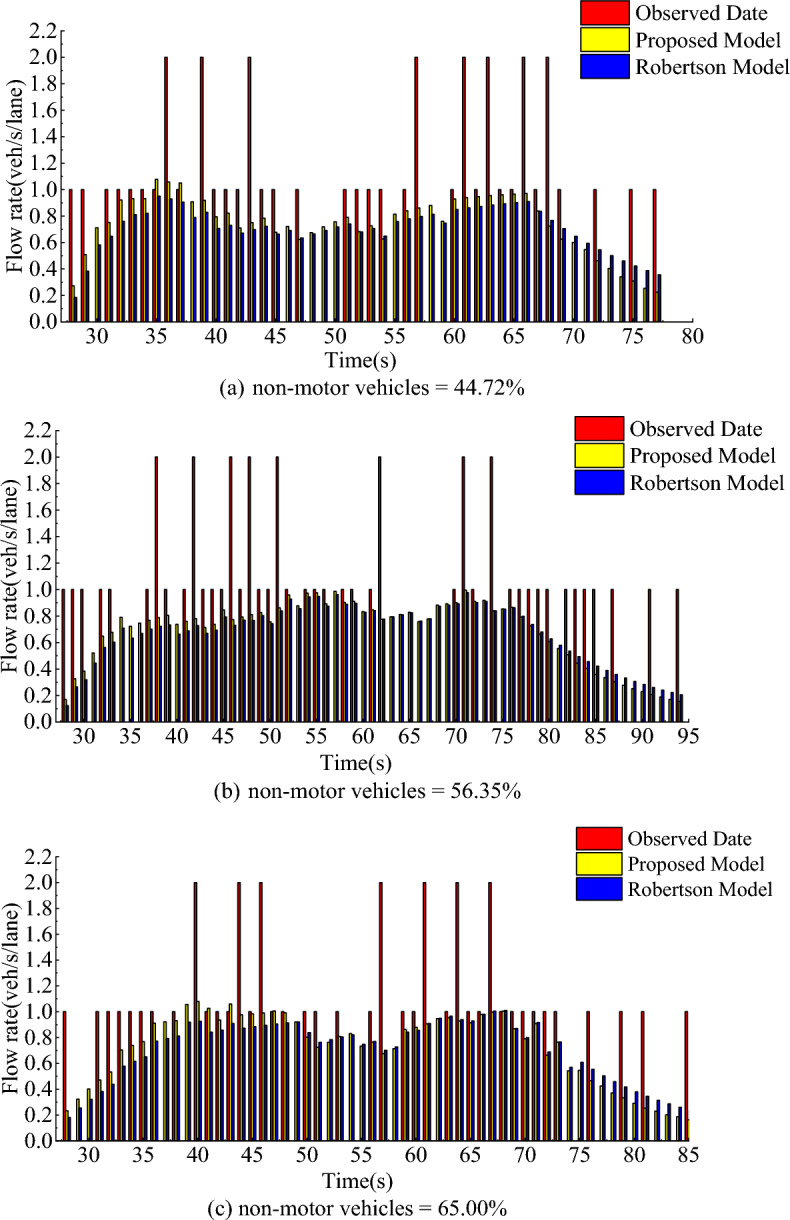
Comparison of arriving flow rate distribution at downstream section.

Through the analysis of Figs. 9 and 10, Tables 1 and 2, it can be seen that:

1. In Fig. 10, the flow distribution of downstream intersection predicted by this model is very similar to the actual observed flow distribution trend, and the error is small. Therefore, the predicted data are very consistent with the field observation data.
2. From the travel time data in Table 1 and the flow profile of the middle and lower reaches in Fig. 10, it can be seen that when the proportion of non-motor vehicles is high. The flow distribution of downstream arrival shows a bimodal feature, which shows that the model can well reflect the characteristics of heterogeneous traffic flow.
3. It can be seen from Fig. 10 that compared with the Robertson model, the model in this paper is closer to the actual arrival rate distribution. According to Table 2, compared with the Robertson model, the average mean square error of the three groups of cases in this model is reduced by 5.89%, indicating that the model can better reflect the characteristics of mixed traffic flow.

## Conclusions

In this study, Robertson's fleet dispersion coefficient under different proportions of non-motorized vehicles was calibrated by using the actual traffic data collected in the field, and the fleet dispersion model in the mixed environment was established. In the selected survey sites in this paper, Zitai Road in Kunming has good characteristics of mixed traffic and non-motorized vehicle traffic. The results show that the calibrated discrete fleet model is reliable, and the proposed method can also be applied to other signal intersections with similar conditions. This study summarizes the following conclusions.

Firstly, the proportion of non-motorized vehicles at signalized intersections has a significant impact on the coefficients of Robertson's platoon dispersion model. The relationship between the proportion of non-motorized vehicles and the platoon dispersion coefficient can be expressed by polynomial formulas. When the proportion of non-motor vehicles increased from 33.33 to 66.70%,$\alpha$ will continue to increase, but when the proportion is close to 50%, it will begin to decrease; the trend of $\beta$ is inversely proportional to $\alpha$, first decreasing, but when the ratio is close to 50%, it gradually begins to increase; as a smoothing coefficient, the change trend of $F$ is similar to $\beta$.

Secondly, the calibration values of $\alpha$,$\beta$ and $F$ are also different from the default values recommended in the TRANSYT user guide. Th calibration value $\alpha$ is greater than the default value of 0.25, fluctuating between 0.25 and 0.4. The calibration value $\beta$ is generally less than the default value of 0.8, fluctuating between 0.7 and 0.8.

Finally, the proposed calibration method can effectively improve the accuracy of the fleet discrete model. Compared with the Robertson model, the prediction error of the proposed model is reduced by 5.89%, which is essential for the optimization of traffic signal control.

Since this paper focuses on the field data collected in Kunming, the research has certain limitations. The influence of other traffic conditions, the relationship between the parameters of the fleet discrete model and traffic conditions, and the application of the fleet discrete model in signal timing planning can be considered. Therefore, further research can consider other factors, such as the study of different lane number, lane width and road length of the non-hybrid fleet discrete model. In addition, on the basis of the improved discrete vehicle fleet model, future research can also optimize the signal timing to reduce the delay caused by the queue length and vehicle-bicycle conflicts at the intersection, so that the model can better serve road traffic in developing countries.

## Data Availability

All data generated or analysed during this study are included in this published article.
